# The Behavior of Organization in Economic Crisis: Integration, Interpretation, and Research Development

**DOI:** 10.1007/s10551-021-04928-8

**Published:** 2021-09-03

**Authors:** Vojko Potocan, Zlatko Nedelko

**Affiliations:** Faculty of Economics and Business, University of Maribor, Razlagova 14, 2000 Maribor, Slovenia

**Keywords:** Economic crisis, Ethical behavior, Unethical behavior, Organizational behavior

## Abstract

We investigated the significance of an economic crisis for organizations’ ethical behavior, employees’ unethical behavior, and association. To capture the effect of the “2008’ World economic crisis,” we compared the behaviors of organizations and employees’ unethical behavior during a crisis with their behavior in more favorable circumstances before and after the crisis. We used structural equation modeling to analyze answers collected from 2024 employees in Slovenian organizations between 2006 and 2016. The results showed significant growth of organizational engagement in ethical behavior, despite the crisis in the middle of the observed period. The employees’ unethical behavior was significantly less acceptable in crisis compared to before the crisis, while after the crisis, its acceptability increased again, despite not significant. The aggregate sample revealed a significantly negative influence of employee’s unethical behavior on organizations’ ethical behavior that was not significantly different across the considered periods. The research suggests the need to manage the organization’s ethical behavior in times of economic downturn, like in the present COVID 19. Additionally, managers need to devote more attention to prevent employees’ unethical behavior and its influence on organizations’ ethical behavior.

## Introduction

Several social, political, and health crises aggravate the society’s workings (Rosenberg, 2012) and lead to the rise of economic crisis (Crescenzi et al., 2016; Rosenberg, 2012). The academic community is becoming increasingly aware that each crisis worsens economic circumstances in society (Christensen & Kohls, 2003; Lins et al., 2017), but less is known about how economic crises affect organizations’ behavior (Donaldson, 2012; Lewis et al., 2010).

In the last decade, social studies have highlighted the importance of behavior in organizations (Kish-Gephart et al., 2019; Trevino et al., 2008), which led to the emergence of studies that explored ethical behavior in organizations in light of the development of societal factors—like culture, values, and education (Gelfand et al., 2017; Goodpaster, 1992). The effect of the crisis on the behavior of organizations was conceptualized in the 1980s (Christensen & Kohls, 2003; Donaldson, 2012) based on Negandhi (1983) conceptualization of situational macro-level factors, like development, political factors, and economic factors, as behavior predictors of fluctuations in organizational behavior in modern society.

At the same time, increasing organizations’ interest in social demands (Rupp et al., 2011; Trevino et al., 2014) revealed the need for multilevel analysis of organizations’ ethical behavior (Brenkert, 2010; Wang et al., 2016). Following the work of Goodpaster (1992), which conceptualized behavior at micro-, mezzo- (often considered as organizational level), and macro-levels and specified individuals, organizations, and society as the main subjects, most recent ethical literature has focused primarily on studying behaviors at the organization level (Gelfand et al., 2017; Glavas, 2016) while paying less attention to organizational relationships with individuals and society (Goodpaster, 1992; Wang et al., 2016).

Perhaps due to the above-mentioned debates, behavior studies have evidenced a significant shift from examining organizational behavior as a multiple social dimension to considering the behavior of single social activities (Jones & Kavanagh, 1996; Rupp et al., 2011). Many scholars have focused their attention on employees’ behavior in the workplace, working relations, and environmental initiatives (Schmidt & Kipnis, 1984; Umphress et al., 2010), among others. However, scholars have not addressed unethical behavior of employees, including the conflict of interest, accounting irregularities, theft, fraud, waste, abuse, and discrimination, in organizations (Robinson and Grenberg, 1998; Kang, 2008) and management of such behavior in changing circumstances in society (Kaptein, 2008; Weber et al., 2003).

Individuals’ unethical behavior varies, and the influence of their behavior on organizational behavior varies as well (Umphress et al., 2010; Weber et al., 2003). Although conceptual studies have addressed the significance of employees’ behavior in organizational behavior (Goodpaster, 1992; Robinson & Greenberg, 1998), they lack empirical evidence on this significance in changeable—trying and favorable—economic circumstances over a longer period (Kaptein, 2010; Trevino et al., 2008).

The present study had empirical and conceptual objectives to advance organizations’ ethical behavior literature. First, we conceptualized organizations’ behavior as ethical and unethical to empirically test the integrality of previous study findings on organizations’ various behaviors.

Second, we complement previous organizational behavior studies by considering three gaps in multilevel consideration of organizational behavior. We complemented previous works with research on how organizational and social norms define the assessment of behavior in organizations (Deshpande, 1997; Jackson, 2001). Next, we contributed to previous research by analyzing employees’ behavior in working relations as a single social activity in organizations, according to ethical studies in the early 2000s (Gelfand et al., 2017; Jacobs et al., 2014). We add to the knowledge of organizational behavior by analyzing how cross-sectional and longitudinal changes in macro-level ethical predictors distinguish ethical behavior in organizations. This opens up a context that allows us to analyze behavior in organizations in pre-crisis, crisis, and after the economic crisis and the influence of individuals’ unethical behavior on organizational ethical behavior in each considered period, as our unique contribution to behavior research.

Fourth, we analyzed the influence of changing economic circumstances on the longitudinal development of behavior in organizations. Our empirical goal was to assess the relative contribution of specific economic circumstances of the “2008 World economic crisis” to predicting organizational behavior differences. This crisis’s real effect on organizations’ ethical behavior has not been explained enough and empirically verified up to now (Markovits et al., 2013; Potocan et al., 2019). Therefore, we investigated ethical behaviors in organizations in the economic crisis and compared them with those occurring in more favorable periods before and after the economic crisis, as our next unique contribution to behavior research.

In the following sections of this paper, we first discuss selected issues of organizations’ ethical behavior in general, followed by Slovenian organizations’ ethical behavior. We justify our selection of organizations and sampled period. Second, we present the analysis of organizations’ ethical behavior, employees’ unethical behavior, and their correlations. Additionally, we compare these behaviors in crisis with behaviors in more favorable circumstances. We conclude the paper by discussing study results, highlighting future research directions and organizational practice implications.

## Theory and Hypotheses

An overview of ethical behavior’s evolution based on a comprehensive, although nonexhaustive literature review can be found in Table 1. Moreover, Table 1 highlights studies that presented origins and established standards of organizational ethical behavior. Our research outlined the selected theoretical basis that informs why and how organizations’ behavior can be researched.

**Table 1:** Overview of selected studies that established a domain of organizational ethical behavior in our research

Study	Focusing on research	Contribution to ethical behavior	Researched issues
Development of ethical behavior	Development of ethical behavior, assessment of behavior ethicality, and conceptualizations of ethical behavior	Conceptualization of ethical responsibility of organizations according to their social norms and broader society norms	Ethical behavior (Gelfand et al., 2017; Trevino et al., 2008) Behavior in organizations (Jackson, 2001; Moore et al., 2012) Employees’ behavior (Kaptein, 2010; Umphress et al., 2010) Methodological issues in research of ethical development (Kish-Gephart et al., 2019; Paterson & Huang, 2019)
Societal factors of ethical behavior	Social circumstances and their relationships with the ethical behavior of organizations	Importance of societal factors for the development of organizational behavior and ways of their influences on the behavior of organizations	National culture (Schwartz, 1992; Blodgett et al., 2001) Level of economic development (Egri & Herman, 2000; Furrer et al., 2010) Legislation (Donaldson, 2012; Jacobs et al., 2014) Characteristics of organizations (Deshpande, 1997; Lins et al., 2017) Employees demographics characteristics (Deshpande, 1997; Kaptein, 2008)
Situational (macro-level) factors of ethical behavior	Characteristics of situational (macro-level) factors and their relationships with the ethical behavior of organizations	Importance of society’ situational circumstances for the development of organizational behavior and ways of their influences on the behavior of organizations	Economic circumstances (Egri et al., 2000; Negandhi, 1983) Changing economic circumstances and behavior of organizations (Christensen & Kohls, 2003; Kaptein, 2010) Favorable and trying economic circumstances and behavior of organizations (Jackson, 2001; Lewis et al., 2010)
Framework for consideration of ethical behavior in	A contextual and methodological framework for consideration of ethical behavior in organizations	Conceptualization of internal and external factors which define consideration of ethical behavior in organizations	Levels of ethical analysis (Goodpaster, 1992; Trevino et al., 2008) Ethicality of working (Mauro, 1995; Tenbrunsel & Smith-Cove, 2008; Remisova et al., 2019) Sustainable development and behavior (Egri & Herman, 2000; Williamson et al., 2006) Behavior as multi-social dimensions and single social activity (Gelfand et al., 2017; Wang et al., 2016) Significance of different level of behavior for organizational behavior (Paterson & Huang, 2019; Umphress et al., 2010)
Classification of ethical behavior	Conceptualization of ethical behavior	Conceptualization of ethical and unethical behavior of organizations according to valid social norms	Bases for classification of ethical behavior Trevino et al., 2008; (Goodpaster, 1992; Trevino et al., 2008) Classification of behavior in organizations (Egri & Ralston, 2004; Trevino et al., 2014)
Economic crisis	Nature and characteristics of economic crises and their influences on the behavior of organizations	Completion of knowledge about behavior in economic crises and specifics of behavior in 2008 World economic crisis	Economic crises in the 1980s (Christensen & Kohls, 2003; Donaldson, 2012) 2008 World economic crisis (Crescenzi et al., 2016; Rosenberg, 2012) Slovenian context of 2008 crisis (Potocan et al., 2019; Pučko, 2000), International context of 2008 crisis (Jaffe & Tsimerman, 2011; Markovits et al., 2013; Soulsby et al., 2017)
Future directions and research opportunities	Unconsidered and less explained areas and levels of ethical behavior	Comprehensive conceptualization of ethical behavior – especially related to situational consideration	Development of qualitative research methods (Wang et al., 2016) Multilevel analysis of organizational behavior (Moore et al., 2012; Kish-Gephart et al., 2019) Sustainable goals and organizational behavior (Gelfand et al., 2017; Williamson et al., 2006) Research of ethical process in organizations (Donaldson, 2012; Kaptein, 2010) Individual predictors of organizational ethical behavior (Robinson and Greenber, 1998; Kish-Gephart et al., 2010)

### Ethical Behavior of Organizations

The theoretical basis presented in Table 1 and results of previous studies on the development and present state of organizations’ ethical behavior (Blodgett et al., 2001; Soulsby et al., 2017) emphasize the need for complementary studies on organizational behavior in a changeable environment.

Previous studies have implemented different organizational and outer social norms in assessing organizational behavior, yielding inconsistent results (Egri et al., 2000; Ralston et al., 2009b). An organization’s social norms that are based on society’s ethical goals (Brenkert, 2010; Gelfand et al., 2017) ensure ethical behavior (Egri et al., 2000; Trevino et al., 2014). Conversely, we considered behavior that is prohibited by valid norms and legislation or opportunistic behavior that hurts organizations as unethical (Mauro, 1995; Robinson & Greenberg, 1998). Such a definition of behavior could be opening up questions regarding the importance of different social norms in assessing behavior in organizations (Gelfand et al., 2017; Jackson, 2001).

For multilevel analysis of the ethical behavior of organizations, we outlined three ethical levels depending on whether research of organizational ethical behavior focuses on the organization itself or personal and social level that can affect or be affected by organizational behavior, referred to as the business context of behavior (Goodpaster, 1992; Rupp et al., 2011). Much of the behavior studies have focused on an organizational level (Blodgett et al., 2001; Christensen & Kohls, 2003); some of them examined mutual relationships between society and organizational behavior (Giannarakis & Theotokas, 2011; Lewis et al., 2010), while fewer studies have addressed relationships between an organization and micro-level behavior (Hagan & Jo Long, 2005). In addition, relationships between an organization and an individual have reflected mainly the organization’s effect on employee behavior (Gelfand et al., 2017; Glavas, 2016). The effects of employees’ behavior have been less researched (Kish-Gephart et al., 2010; Trevino et al., 2008). Therefore, we examined the effects of the unethical behavior of employees on organizational ethical behavior.

Finally, in the last decade, scholars have highlighted the need to discover the cause-and-effect relationships between changes in macro-level circumstances in society and fluctuations in organizational ethical behavior (Donaldson, 2012; Lins et al., 2017). Such a research focus has revealed that analyzing only one kind of circumstance in society neglects important circumstance variations that would more fully explain behavior in an organization (Markovits et al., 2013). To integrate the presumptions mentioned above, we created a comparative analysis of organizational behavior in economic crises and periods with more advantageous economic circumstances.

### Ethical Behavior of Slovenian Organizations

We selected Slovenian organizations that were affected by equal societal characteristics (Mrak et al., 2004; Potocan et al., 2019). The ethical behavior of sampled organizations before Slovenia joined the European Union in 2004 was influenced by socialist systems in ex-Yugoslavia in the period from 1945 to 1991 and the democratic parliamentary system in an interdependent state after 1991 (Jelovac et al., 2011; Potocan et al., 2019).

Ethical behavior of organizations in ex-Yugoslavia reflected primarily Central European cultural tradition of the socialist political system, “self-management” governance of organizations, and development of Slovenian organizations’ market orientation after 1970 (Lang et al., 2013; Mrak et al., 2004). The period from independence in 1991 and the starting of joined negotiations with the European Union (EU) in 1998 denoted the transition process from a socialist economic system to the modern market economy together with the development of values systems, culture, and ethic of organizations according to EU behavior norms and standards (Mrak et al., 2004). Additionally, organizations have gone through structural restructuring accompanying “wild privatization,” disregarding social norms and growing social inequality (Lang et al., 2013; Mrak et al., 2004). During membership negotiations with the EU in the period from 1998 to 2004, organizations standardized their behavior based on the EU’s economic, social, and ethical principles and legislation (Jelovac et al., 2011). The latest studies on behaviors of Slovenian organizations from this period noted the weakness of the “ethical legacy of socialistic and transition periods” (Banalieva et al., 2017; Lang et al., 2013) and their successful implementation of advanced EU’ ethics in business practice (Lang et al., 2013; OECD, 2018).

Slovenian societal development after joining the EU in 2004 reflected the reconciliation of Slovenian and EU development (Lang et al., 2013; Reynaud et al., 2007) and followed the development of ethical behavior on common social norms, ethical framework, and standards of EU (Jelovac et al., 2011; OECD, 2018). The main reason for changes in the ethical development of macro-level factors in EU societies in the last decades caused the formation of unique organizations’ ethical behaviors (Crescenzi et al., 2016; OECD, 2018). Based on macro-level factors exposed in the literature (Negandhi, 1983; Ralston et al., 2009b), we directed our attention to economic circumstances and analyzed the relationships between economic circumstances and Slovenian organizations’ ethical behavior from the year 2006 to the year 2016. In 2006, Slovenian organizations ended their implementation of EU regulations and standards (Jelovac et al., 2011; Potocan et al., 2019), and in 2016, the sampled organizations were recovering from the consequences of the “2008 World economic crisis” (Strazisar et al., 2016).

During the considered 10-year period, major changes in economic circumstances caused by the “2008 World economic crisis” that started in the USA as the financial crisis in 2008 (Rosenberg, 2012) hit the European economy in 2009 (Crescenzi et al., 2016) and affected Slovenia “from the 3rd quarter of 2009 to the 1st quarter of 2013” (OECD, 2018, p. 15). This economic crisis affected Slovenian organizations through declining of domestic consumption and the slowdown of exports that resulted in the initial shrinkage of the national GDP by 7.9% in 2009, which was the third biggest fall in the EU, and a drop of 12.50% GDP during the entire period of crisis (Strazisar et al., 2016). From the 2nd quarter of 2013 onwards, Slovenia’s GDP started rising again (Strazisar et al., 2016).

Slovenia’s media exposed the worsening of organizations’ behavior in this crisis (Potocan et al., 2019) caused by “business scandals” and “interference of the political sphere in corporate governance of organizations.” Additionally, scholars reported on the enlargement of organizations’ unethical practices, like accounting irregularities, discrimination, and harassment (Lang et al., 2013) and the increase in negative behavior among employees, like misusing company time and abusive behavior and theft (Jelovac et al., 2011). However, this crisis’s effects on Slovenian organizations’ behavior and their stakeholders were not explained or empirically tested enough up to now.

To consider the behavior of organizations in crisis and more favorable economic circumstances, we divided the selected period into three sub-periods characterized by (1) advanced economic circumstances in the period from 2006 to 2008, considered as the period before the crisis, (2) worsening of economic circumstances during “2008 World economic crisis”, considered as the period of crisis, and (3) recovering of economic circumstances after “2008 World economic crisis” from 2013 to 2016, considered as the period after the crisis.

### Development of Research Hypotheses

#### Ethical Behavior of Organizations

Theoretical foundations for considering outer social norms in organizations originated in studies on the importance of sustainable development and environmental and social goals for organizations in the 1980s (Deshpande, 1997; Jones & Kavanagh, 1996), which were verified in later empirical studies (Egri & Herman, 2000; Ryu, 2019; Williamson et al., 2006).

Major research has reported increasing acceptance of social norms in organizations as a general behavioral trend (Moore et al., 2012; Tenbrunsel & Smith-Cove, 2008). Still, inconclusive results were found regarding the effect of worsening economic circumstances on the fluctuations in organizations’ behavior (Jackson, 2001; Steurer & Konrad, 2009). Several studies in the 1980s and 1990s reported that economic crises decrease ethical behavior (Christensen & Kohls, 2003; Hagan & Jo Long, 2005) while newer studies, mainly related to the “2008 World economic crisis,” have not reported a large negative influence of this crisis on the behavior of organizations (Johnson, 2018; Wang et al., 2016). Behavior studies on the aforementioned crisis have noted that organizations increased the adoption of legislative and outer societal norms to retain and recover favorable business outcomes (Giannarakis & Theotokas, 2011; Markovits et al., 2013). Scholars have also reported the growing engagement of organizations in ethical behavior during the crisis (Gelfand et al., 2017; Trevino et al., 2014) caused by demanding ethical expectations and higher sensitivity to social issues (Kish-Gephart et al., 2019; Lins et al., 2017). The above-mentioned studies have supported our presumption that the circumstances of the “2008 World economic crisis” intensified interest and engagement of organizations in ethical behavior.

Studies addressing the prediction of organizational ethical behavior in favorable circumstances stated its long-term progress initiated by advanced societal norms (Blodgett et al., 2001; Moore et al., 2012). Other scholars have added knowledge about the importance of perception of circumstances (Jackson, 2001) and interests in ethical behavior (Egri et al., 2000; Rupp et al., 2011) in the formation of organizations’ behavior. On the contrary, individual studies have indicated a decrease in organizational adoption of valid outer social norms in favorable circumstances (Furrer et al., 2010; Lang et al., 2013) and explained that organizations might pay less attention to the achievement of outer societal norms in time of economic prosperity (Jones & Kavanagh, 1996; Paterson & Huang, 2019). The above-mentioned studies have supported our presumption that organizations care less about achieving outer social norms in favorable circumstances.

Integration of studies on the long-term advances of ethical behavior in organizations (Egri & Herman, 2000; Trevino et al., 2014), studies on the larger enhancement of organizational ethical behavior in crises (Giannarakis & Theotokas, 2011; Markovits et al., 2013), and surveys of behavior in organizations during the “2008 World economic crisis” (ERC, 2009; WEF, 2010) guided our analysis of ethical behavior of organizations during the considered periods. Thus, we hypothesized:

##### H1

Engagement of organizations in ethical behavior will be higher in a period of economic crisis compared to periods before and after the economic crisis.

#### Employees’ Unethical Behavior

We continued to analyze how the behavior of employees reflected organizational ethical norms linked to working relations. Ethical studies in the 1980s conceptualized the importance, extent, and societal damage of unethical behavior in organizations (Deshpande, 1997; Robinson & Greenberg, 1998), whereas further studies in the 1990s expanded the existing knowledge of the prevailing influence of situational circumstances on behavior in organizations (Kish-Gephart et al., 2010; Weber et al., 2003). Thus, studies have reported that the worsening of situational circumstances increases unethical behavior of employees (Umphress et al., 2010) while improvements in circumstances reduce it (Deshpande, 1997). In addition, behavior studies on economic crises in the 1980s revealed that these crises encouraged unethical practices among employees (Christensen & Kohls, 2003; Robinson & Greenberg, 1998).

Scholars in the 2000s expanded the discussion of unethical behavior with the conceptualization of personal behavior factors, such as moral development, beliefs, values, attitudes, and their relationships with the actual behavior of individuals in specific circumstances (Glavas, 2016; Paterson & Huang, 2019). For instance, individuals can react to worsening circumstances with less or more ethical behavior according to their perceptions of suitable behavioral responses to such circumstances (Egri & Herman, 2000; Robinson & Greenberg, 1998), which may explain the differences in employees’ unethical behavior during the economic crises in the last decades. According to the “2008 world economic crisis,” Jaffe and Tsimerman (2011) noted that employees’ unethical practices in organizations did not increase when international behavior surveys from the same period reported lower acceptability of unethical behavior and practices among employees and organizations (ERC, 2009; WEF, 2010). Such fluctuations in behavior may explain employees’ tendency to engage in unethical behavior to avoid risk during the time of high uncertainty (Blodgett et al., 2001; Kish-Gephart et al., 2010) as well as advanced ethical culture and higher ethical standards of their organizations (Lins et al., 2017; Tenbrunsel & Smith-Cove, 2008).

Attempts to explain the relationships between favorable circumstances and employees’ unethical behavior revealed even more contradictory results (Gelfand et al., 2017; Trevino et al., 2014). Scholars have presumed decreased organizations’ acceptance of employees’ unethical behavior in such circumstances because of their active efforts to reduce unethical actions, behavior, and practices (Furrer et al., 2010; Markovits et al., 2013). More recent studies have mentioned examples of higher acceptability of such behavior in organizations (Gelfand et al., 2017; Paterson & Huang, 2019), possibly due to decreased attention to inappropriate behavior in prosperity times (Mauro, 1995; Ramus & Steger, 2000).

The aforementioned studies of unethical behavior of employees in crisis and favorable circumstances (Mauro, 1995; Paterson & Huang, 2019) and surveys about the behavior of employees in considered crisis (ERC, 2009; WEF, 2010) guided our analysis of employees’ unethical behavior regarding promotion at the workplace under changing economic circumstances. We, therefore, proposed the following:

##### H2

Employees’ acceptability of unethical behavior will be lower in the period of economic crisis than in periods before and after the economic crisis.

#### The Effect of Employees’ Unethical Behavior on the Ethical Behavior of Organizations

We concluded our study by analyzing the significance of employees’ unethical behavior for organizational behavior. We first tested this significance for each considered circumstance separately, followed by analyzing the significant fluctuations in changing circumstances over the ten years.

Researchers before the 2000s reported that ethical or unethical behavior has the same significance for organizational behavior in diversified circumstances (Mauro, 1995; Umphress et al., 2010). Such presumption supported studies on sustainable development (Egri & Herman, 2000; Williamson et al., 2006). Later, scholars have expanded this significance with presumptions about oscillations of employees’ behavior in changing situational circumstances (Robinson & Greenberg, 1998; Weber et al., 2003). For instance, Weber et al. (2003) revealed an increasing effect of employees’ theft in a difficult period on organizations’ stability, while Christensen and Kohls (2003) noted the growing significance of employees’ ethical behavior for organizational decision-making in a business crisis.

Studies on employees’ behavior during the “2008 world economic crisis” added new insights into this issue (Johnson, 2018; Paterson & Huang, 2019), showing decreased employees’ unethical behavior in this crisis (Giannarakis & Theotokas, 2011; Jaffe & Tsimerman, 2011). Additionally, studies on employees’ engagement in improvement of their behavior in severe situations (Lins et al., 2017; Markovits et al., 2013) and surveys indicating lower benevolence of employees to unethical behavior (ERC, 2009; WEF, 2010) have also supported presumptions about the decreased significance of employees’ unethical behavior for organizations.

Consistent with studies on the significance of employees’ unethical behavior in different circumstances (Robinson & Greenberg, 1998; Weber et al., 2003), reduced employees unethical behavior in crisis (Jaffe & Tsimerman, 2011; Markovits et al., 2013), and the significance of employees behavior for organizations behavior in the “2008 world economic crisis” (Paterson & Huang, 2019), we formed the following hypothesis:

##### H3

The effect of employees’ unethical behavior on organizational ethics will be weaker during the crisis than in the period before and after the crisis.

## Methods

### Instrument

We have been involved in “The University Fellows International Research Consortium” (University of Oklahoma)” for the last two decades. For the “Consortium,” we collected data in Slovenian organizations every five years using a “Consortium questionnaire.” “Consortium questionnaire” has been validated in numerous international cross-cultural studies (Egri & Ralston, 2004; Furrer et al., 2010; Ralston et al., 2009b), including studies in Slovenia (Banalieva et al., 2017; Potocan et al., 2019; Reynaud et al., 2007).

A Slovenian version of the questionnaire used in this research was derived from the original questionnaire developed by the “The University Fellows International Research Consortium.” In 2005, the entire “Consortium original questionnaire” was translated into Slovenian language, but we included the 25 listed items of activities in the third part.

With the Consortium’s permission, we refocused our attention from measuring employees’ attitudes to organizational engagement in corporate social responsibility (CSR) to measure employees’ perceptions of whether their organization behaves according to the goals of the listed activities. Therefore, the introduction text in part 3 can be read (with the English translation of Slovenian’ meaning of the text) as follows: instructions—the following text lists various activities that organizations can do. “We are interested in your opinion on whether your organization behaves according to the listed activities’ goals”. We gathered data with this instrument yearly from 2006 on. Every fifth year, we added the original third part to the Slovenian version of the questionnaire for the consortium’s purposes. In this research, we used data from the Slovenian version of the questionnaire, including 2006 to 2016. We used the data from the second, third, and fourth parts***.***

The first part of the questionnaire included 56 personal values adopted from the Schwartz value survey (Schwartz, 1992). The second part included 38 short scenarios regarding possible strategies for advancement in the workplace (Egri et al., 2000). The third part consisted of 25 items measuring participants’ opinions about the organization’s behavior according to listed activities (Ralston et al., 2015; Trevino et al., 2014). The fourth part consisted of ten personal and organizational demographic variables typically used in business studies (Egri & Ralston, 2004; Ralston et al., 2009b). We used the data from the second, third, and fourth parts*.*

The data collection process from 2006 followed the same guidelines each year. According to the European NACE Rev. 2 classification of the industry, the sample should have reflected the Slovenian industry structure, which we transformed according to the USA’s NAICS classification. By collapsing 22 industries into ten broader industry categories, we reduced organizations’ fragmentation across numerous industries. Second, we kept the original classification of organizational size, with small organizations having below 100 employees, medium organizations having between 100 and 1000, and large organizations having more than 1000 employees. Organizations with fewer than 50 employees dominate the Slovenian economy (> 90%) and play an important role in economic development (Bartlett & Bukvič, 2001). Additionally, we limited the inclusion of respondents from small organizations to 50% of the sample. Medium organizations were limited to 30% of the sample, while large organizations were limited to 20%. Third, to reflect the ratio between the manufacturing and service organizations, we included up to 40% of respondents from manufacturing organizations. Finally, we tried to obtain at least 150 answers to have enough cases for analyses and ensure our data’s representativeness.

Following the above-mentioned guidelines, each year, we contacted 1000 employees in Slovenian organizations while collecting a maximum of two answers per organization. Organizations were selected from Slovenian companies’ official BISNODE directory using a random number generator in MS Excel. Each year, different respondents participated in the survey. Organizations were first contacted via phone. After agreeing to participate in the research, the selected participants were contacted via phone (computer-assisted telephone interviews—CATI) to gather their responses.

### Sample

Sample comprised 2024 employees’ responses collected in 2006 (*n* = 225) (response rate 22.5%), 2007 (*n* = 224) (22.4%), 2008 (*n* = 220) (22%), 2010 (*n* = 182) (18.2%), 2011 (*n* = 183) (18.3%), 2012 (*n* = 247) (24.7%), 2013 (*n* = 184) (18.4%), 2014 (*n* = 182) (18.2%), 2015 (*n* = 184) (18.4%), and 2016 (*n* = 193) (19.3%). The year 2009 was excluded because the crisis started in 2009, and the results of wave-analysis showed divergent results for the first and second waves. The characteristics of aggregated sample (*n* = 2024) are outlined in Table 2.

**Table 2:** Demographic characteristics of respondents

Characteristic	Aggregated sample (*n* = 2024)
Age	41.67
Gender	
Male	41.8%
Female	58.2%
Education	
Finished secondary school	20.6%
Finished a bachelor’s degree	60.0%
Finished master’s degree	17.3%
Finished a doctoral degree	2.2%
Position in organization	
Non-managerial staff	46.9%
First-level manager	25.1%
Mid-level manager	20.8%
Upper-level manager	7.2%
Organization size	
Fewer than 100 employees	49.2%
100 to 1000 employees	34.6%
More than 1000 employees	16.2%
Industry of organization	
Manufacturing	
Agriculture, mining, forestry, fishing	2.6%
Construction	2.5%
Production	25.3%
Service	
Transportation, communication, utilities	17.8%
Wholesale and retail trade	14.2%
Finance, insurance, real estate	15.0%
Services	6.6%
Public administration	13.9%
Health care	2.0%
Other	0.1%

### Measures

#### Employees’ Unethical Behavior

We used 38 short scenario items validated in several cross-cultural studies (Egri et al., 2000; Karam et al., 2013; Ralston et al., 2009a) to assess respondents’ views of the ethicality regarding promotion in the workplace.

The scenarios measure several ethical and unethical strategies that individuals might use to try to get ahead at work. Each statement started with, “As a strategy to get ahead at my work, my co-workers would consider it acceptable [ethical] to …”. Participants rated statements on an 8-point scale ranging from 1 (extremely acceptable) to 8 (extremely unacceptable). Asking sensitive questions about the individuals’ unethical behavior may enhance social desirability bias (Umphress et al., 2010). Peer-assessment reduces social desirability bias and the temptation to provide desirable responses when asking about sensitive topics (Trevino et al., 2008; Zuber & Kaptein, 2014). Several cross-cultural studies applied these 38 short scenarios in peer assessment (Egri et al., 2000; Karam et al., 2013; Ralston et al., 2009a), as it is the case in our research. Confirmatory factor analysis (KMO = 0.953, Bartlett’s test of sphericity = 33,451.54; df = 703; *p* < 0.001) revealed a four-factor structure, namely organizationally sanctioned behavior, non-destructive/legal behavior, destructive/legal behavior, and destructive/illegal behavior, replicating the results of prior studies using 38 scenarios (Egri et al., 2000). In line with this research aims, we focused on destructive behavior factors to address the employees’ unethical behavior. We formed two variables, namely destructive legal behavior (EUB 1, 3, 5, 6, 9, 10, 13, 14) (Cronbach’s *α* = 0.886) and destructive illegal behavior (EUB 2, 4, 7, 8, 11, 12, 15, 16) (Cronbach’s *α* = 0.894). Both variables were highly correlated (*r* = 0.803), indicating that they essentially. Items measuring both variables also showed significant moderate to strong correlations (from 0.334 to 0.632). Additionally, after establishing a measurement model with two formed variables, modification indices in AMOS suggested adding correlations between items of both variables. Since both variables were highly correlated and measured the same phenomenon, i.e., unethical behavior, we combined them into one variable (Ho, 2006). Finally, we formed variable employees’ unethical behavior consisting of 16 items, with 8 items measuring destructive legal behavior and 8 items measuring illegal behavior, as identified in a prior study (Egri et al., 2000). Details about factor loadings, average variance extracted (AVE), composite reliability (CR), and Cronbach’s *α* coefficients for formed construct is outlined in Table 3.

**Table 3:** Latent variables, measurement items, factors loadings, AVE, CR, and Cronbach’s *α*

	Factor loadings	AVE	CR	Cronbach’s *α*
Employee’s unethical behavior—EUB		0.454	0.930	0.936
“As a strategy to get ahead at my work, my co-workers would consider it acceptable to …”				
UNEP 1—spread rumors about someone or something that stands in the way of their advancement	0.625			
UNEP 2—hire a criminal to seriously injure a competitor for a promotion	0.566			
UNEP 3—try to influence the boss to make a bad decision, if that decision would help them to get ahead	0.666			
UNEP 4—use detrimental information to blackmail a person who is in a position to help them get ahead in the organization	0.734			
UNEP 5—use their network of friends to discredit a person competing with them for a possible promotion	0.759			
UNEP 6—withhold information to make someone else look bad	0.729			
UNEP 7—put a listening device, such as a tape recorder, in the office of a competitor for a promotion to get information about this person	0.649			
UNEP 8—threaten to give valuable company information to someone outside the organization if their demands are not met	0.674			
UNEP 9—offer sexual favors to a superior	0.627			
UNEP 10—blame others for their own mistakes	0.710			
UNEP 11—try to create a situation where a competitor for a promotion might be caught using illegal drugs or engaging in some other illegal activity	0.584			
UNEP 12—try to get the answers to a job promotion examination to ensure that they would score higher than the others taking the exam	0.672			
UNEP 13—put false information on a job resume to make themselves look better than they are	0.722			
UNEP 14—try to develop contacts who might be able to provide detrimental information about one of their competitors for a promotion	0.732			
UNEP 15—steal secret corporate documents and give them to another company in return for a better job at the other company	0.655			
UNEP 16—make anonymous, threatening phone calls to psychologically stress a competitor for a promotion	0.639			
Ethical behavior of organization—EBO		0.359	0.848	0.820
“My organization behaves according to goals of activity to:”				
EBO 1—prevent environmental degradation caused by the pollution and depletion of natural resources	0.546			
EBO 2—adopt formal programs to minimize the harmful effects of organizational activities on the environment	0.703			
EBO 3—always submit to the principles defined by the regulatory system	0.613			
EBO 4—give priority to ethical principles over economic benefits	0.601			
EBO 5—contribute actively to the welfare of our community	0.616			
EBO 6—devote resources to environmental protection even when economic profits are threatened	0.505			
EBO 7—voluntarily exceed government environmental regulations	0.592			
EBO 8—help solve social problems	0.625			
EBO 9—play a role in our society that goes beyond the mere generation of profits	0.550			
EBO 10—train their employees to act within the standards defined by the law	0.620			

#### Ethical Behavior of Organizations

We used 25 items from the third part of the Slovenian questionnaire in which we asked the respondents to indicate whether they agree or disagree that their organization’s goals of activities which reflect society’s social norms accepted by organizations (Ralston et al., 2015). Each statement started with, “My organization behaves according to goals of activity to …”. Participants rated each statement on a 9-point scale ranging from 1 (strongly agree) to 9 (strongly disagree).

To formulate organizations’ ethical behavior variable**,** we followed Carroll’s theory of four types of CSR (discretionary, ethical, legal, and economic), where we focused on ethical behavior, emphasizing societal moral codes of conduct (Carroll & Buchholtz, 2008). Prior research (Furrer et al., 2010), which used the same 25 items as in our research, revealed two principal factors, ethical responsibility and economic responsibility activities. Accordingly, we conducted an exploratory factor analysis (KMO = 0.896, Bartlett’s test of sphericity = 13,162.76; df = 300; *p* < 0.001), setting the number of factors to extract to two, to distinguish between ethical and economic responsibility. We formed variable ethical behavior of organizations based on the ethical behavior factor that reflects society’s social norms accepted by organizations. The newly formed variable included ten items which representing the environmental (EBO 1, 2, 6, 7) (Furrer et al., 2010; Ralston et al., 2015), the social (EBO 3, 9, 10) (Ralston et al., 2015), and ethical aspect (EBO 4, 5, 8) (Furrer et al., 2010) of CSR. Details about factor loadings, AVE, CR, and Cronbach’s *α* coefficients for the above formed latent variable is outlined in Table 3.

We controlled for several personal and organizational demographic characteristics, namely, respondent’s age, gender, level of education, employee’s position in the organization, the size of the organization, and the industry type according to NAICS, in line with other business studies (see, for example, Ralston et al., 2009b).

Since most variables, except for age and gender, were discrete with more than two categories, we converted them into a set of dichotomous variables by dummy variable coding. The education level was converted into a set of three dummy variables (secondary school vs. non-secondary school, bachelor’s degree vs. non-bachelor’s degree, master’s degree vs. non-master’s degree), setting Ph.D. as a reference category. The organization’s position was converted into a set of three dummy variables (first level vs. non-first level, middle level vs. non-middle level, upper level vs. non-upper level), setting professionals as a reference category. The organizational size was converted into a set of two dummy variables (up to 100 vs. not up to 100, between 100 and 1000 vs. not between 100 and 1000), setting organizations with more than 1000 employees as a reference category. In terms of industry, we collapsed the industries into two clusters: (1) manufacturing 1, 2, 3, and (2) service cluster comprising categories from 4 to 10 (Potocan et al., 2019) to reduce the degrees of freedom and obtain a dichotomous variable.

We also dummy coded the variable “economic circumstances,” where we distinguished between (1) period before the economic crisis (years 2006, 2007, and 2008), (2) period of the economic crisis (years 2010, 2011, and 2012), and (3) period after the economic crisis (years 2013, 2014, 2015, and 2016). Accordingly, we created a set of two dummy variables (pre-crisis vs. non-pre-crisis; after crisis vs. not after the crisis), setting crisis as a reference category.

### Instrument Validation

Concerning the internal validity of the created variables, employee’s unethical behavior and organizations’ ethical behavior were both well above the cut-off point of 0.7 (Nunnally, 1978). The items comprising variable employees’ unethical behavior were verified in numerous studies assessing the ethicality of strategies for advancement at the workplace (Egri et al., 2000; Karam et al., 2013; Ralston et al., 2009a), providing evidence that items used in this study are measuring unethical behavior of employees. Similarly, 25 items assessing CSR have been used in previous CSR studies assessed by the employees (Egri et al., 2000; Ralston et al., 2015). Our study used data expressing survey participants’ perspectives on whether their organization behaves according to CSR’s goals.

Factor loadings for both latent variables ranged from 0.505 to 0.759, which are well above the cut-off value of 0.40 reported in Henson and Roberts’ (2006) research of 37 studies published in four psychological journals.

In terms of convergent validity of measures, the CRs for both latent variables were way above the suggested threshold of 0.600 (Fornell & Larcker, 1981). The AVEs were below the recommended level of 0.500 (Fornell & Larcker, 1981), but according to Fornell and Larcker (1981, p. 46), AVE is a more conservative validity measure. Based on CR alone, the researchers can conclude that the variable’s convergent validity is adequate, despite more than 50% of the variance being due to an error. For instance, Lam (2012) reported AVE above 0.310 as acceptable.

For CR and AVE, we do not have reference values, as studies using an original instrument from Consortium typically did not report CR and AVE values (Egri et al., 2000; Furrer et al., 2010; Potocan et al., 2019; Ralston et al., 2015). Given the absence of those values and studies that established the adequate cut-off points for CR and AVE values (Fornell & Larcker, 1981; Lam, 2012), we can conclude that our measures are reliable.

### Research Design and Analyses

Our research followed the following steps:

- Step 1—We outlined the descriptive statistics and the zero-ordered correlations between the variables of interest for the aggregated sample (*n* = 2024).
- Step 2—We used the analysis of variance (ANOVA) and post hoc comparisons to examine differences in the employees’ unethical behavior and organizations’ ethical behavior before, during, and after the crisis.
- Step 3—We tested the effect of employees’ unethical behavior on an organization’s ethical behavior. First, we used hierarchical regression analysis in SPSS 24 to examine the effects of control variables (age, gender, education, position in the organization, organizational size, and industry), which were entered first in the analysis. Next, we examine the effect of economic circumstances, which were entered second, and finally the effect of employee’s unethical behavior, which was entered third, on ethical behavior of organizations was examined. Since economic circumstances were entered as a predictor variable, we tested the effect of employee’s unethical behavior on the organization’s ethical behavior using structural equation modeling in AMOS 24 to determine their effects in crisis as well as before and after the crisis. Finally, we calculated 95% confidence intervals of standardized beta coefficients using bootstrapping in AMOS to differences between obtained beta coefficients across three observed periods. We followed the suggestions of Cumming (2009), who claimed that significant differences exist between beta coefficients when the corresponding 95% confidence intervals overlap by not more than 50%.

Since the source of all variables was in one instrument, common method bias could have confounded our results (Podsakoff et al., 2012). First, we estimated the common method variance utilizing an exploratory factor analysis in SPSS 24. All 38 items from the second and 25 items from the third part of the survey were loaded onto a single factor and constrained without rotation (Podsakoff et al., 2012). The newly introduced common latent factor explained 19.07% of the variance, indicating that the common method bias is way below the threshold value of 50% (Lindell & Whitney, 2001). Next, we used a method that suggests using the marker variable that is theoretically unrelated to the principal variables in the research (Lindell & Whitney, 2001). We used education, which was not significantly related to the concerns about natural, social, and economic resources in the frame of CSR surveys (Furrer et al., 2010; Potocan et al., 2019) and was not emphasized as an important factor in studies on ethical and unethical behavior (Egri et al., 2000; Ralston et al., 2009a). Low correlations between education and two principal constructs in our study (highest was *r* = 0.12) showed no common method bias (Lindell & Whitney, 2001). We can argue that the likelihood of common method bias in this study was low.

The analysis of the multicollinearity between employee’s unethical behavior and the ethical behavior of the organization revealed “tolerance values” ranging from 0.776 to 0.986 and the VIF values ranging from 1.014 to 1.306. Tolerance values greater than 0.10 and VIF values below 10 are acceptable (Hair et al., 1998, p. 14) and indicate that multicollinearity is not an issue in this study.

The goodness of fit of the proposed research model was calculated for the two-factor measurement model that included employees’ unethical behavior and ethical behavior of organization. The fit statistics *χ*^2^ (*N* = 2024, df = 894 = 30,894.27, *p* < 0.001; CFI = 0.910; IFI = 0.911; RMSEA = 0.035; PCLOSE (1.000) indicated a good fit between the hypothesized model and data (Byrne, 2010).

## Results

The means, standard deviations, and correlations among the study variables for the aggregated sample are presented in Table 4.

**Table 4:** Mean values, standard deviations, and correlations among the study variables

Variable	M	SD	1	2	3	4	5	6	7	8	9	10	11	12	13	14
1. Age	41.67	9.18	1													
2. Gender	1.58	0.49	− 0.12***	1												
3. Education—Secondary school	0.01	0.08	− 0.01	0.03	1											
4. Education—A bachelor’s degree	0.20	0.40	0.01	0.03	− 0.04	1										
5. Education—Master’s degree	0.61	0.49	− 0.04	− 0.02	− 0.10***	− 0.64***	1									
6. Position—first level	0.25	0.43	− 0.05*	0.02	0.00	− 0.13***	0.08**	11								
7. Position—middle level	0.21	0.41	0.09***	− 0.05*	− 0.04	− 0.09***	− 0.04	− 0.30***	1							
8. Position—upper level	0.07	0.26	0.10***	− 0.07**	− 0.02	− 0.05*	0.00	− 0.16***	− 0.14***	1						
9. Organizational size—up to 100	0.49	0.50	− 0.15***	0.01	0.01	0.03	0.00	− 0.02	− 0.08**	0.00	1					
10.. Organizational size—between 100 and 1000	0.35	0.48	0.05*	− 0.01	0.01	− 0.02	0.02	0.05*	0.03	− 0.04	− 0.72***	1				
11. Industry	1.70	0.46	− 0.07**	0.10***	0.01	0.04	− 0.01	0.00	0.02	0.01	0.10***	0.01	1			
12 Economic circumstances—pre-crisis	0.30	0.46	− 0.47***	0.14***	− 0.01	− 0.05*	− 0.01	0.07**	− 0.01	− 0.12***	0.15***	− 0.08***	0.02	1		
13 Economic circumstances—after-crisis	0.37	1.38	0.29***	− 0.09***	0.01	− 0.08***	0.00	0.03	0.12***	0.10***	− 0.20***	0.04	0.02	− 0.49***	1	
14 Employee’s unethical behavior	6.51	1.38	0.03	0.04	0.03	0.04	0.00	− 0.02	− 0.03	− 0.02	0.00	0.02	0.00	− 0.09***	0.03	1
15. Ethical behavior of the organization	2.93	1.17	− 0.06**	− 0.01	− 0.01	− 0.03	0.02	0.01	0.06**	0.00	0.06**	− 0.02	− 0.01	0.10***	− 0.10***	− 0.27***

*N* = 2024

*p < 0.05; **p < 0.01; ***p < 0.001

We examined the state of employees’ unethical behavior and organizations’ ethical behavior before, during, and after the crisis (Table 5). Table 6 summarizes multiple comparisons between considered periods for both latent variables to highlight the difference between the three observed periods.

**Table 5:** Employees’ unethical behavior and organizational ethical behavior before, during, and after the crisis

Variables	Before crisis (2006, 2007, 2008)		Crisis (2010, 2011, 2012)		After crisis (2013, 2014, 2015, 2016)		
	Mean	SD	Mean	SD	Mean	SD	F
Employee’s unethical behavior	6.30	1.42	6.62	1.24	6.56	1.46	9.51***
Ethical behavior of organization	3.12	1.07	2.94	1.18	2.79	1.20	13.44***

*p < 0.05; **p < 0.01; ***p < 0.001

**Table 6:** Post hoc comparisons of employees’ unethical behavior and organizational ethical behavior in pre-crisis, crisis, and after the crisis

Dependent variable	(I) Economic circumstances	(J) Economic circumstances	Mean difference (I-J)	Std. error	95% Confidence interval	
					Lower bound	Upper bound
Employee’s unethical behavior	Pre-crisis	Crisis	− 0.32***	0.08	− 0.50	− 0.14
		After-crisis	− 0.25**	0.08	− 0.43	− 0.07
	Crisis	Pre-crisis	0.32***	0.08	0.14	0.50
		After-crisis	0.07	0.07	− 0.11	0.24
	After-crisis	Pre-crisis	0.25**	0.09	0.07	0.43
		Crisis	− 0.07	0.07	− 0.24	0.11
Ethical behavior of the organization	Pre-crisis	Crisis	0.18*	0.07	0.03	0.34
		After-crisis	0.33***	0.06	0.18	0.48
	Crisis	Pre-crisis	− 0.18*	0.06	− 0.34	− 0.03
		After-crisis	0.15*	0.06	0.00	0.30
	After-crisis	Pre-crisis	− 0.33***	0.06	− 0.48	− 0.18
		Crisis	− 0.15*	0.06	− 0.30	0.00

*p < 0.05; **p < 0.01; ***p < 0.001

The results in Table 5 show that the ethical behavior of organizations improved significantly during the observed period, and the results in Table 6 indicate that the ethical behavior of organizations improved significantly from pre-crisis to crisis (0.18; *p* < 0.05) as well as from crisis to after-crisis (0.15; *p* < 0.05). Engagement of organizations in ethical behavior in the crisis was higher than before the crisis but lower than after the crisis, refuting Hypothesis 1.

Further, the results in Table 5 show that the acceptability of employees’ unethical behavior in a crisis was lower than before and after the crisis. Additionally, the results in Table 6 indicate that the acceptability of employee’s unethical behavior decreased significantly from pre-crisis to crisis (− 0.32; *p* < 0.001), while from crisis to after the crisis, its acceptability increased again, despite not significant (0.07; *p* > 0.05). These findings provide support for Hypothesis 2.

Next, we outline the effect of employee’s unethical behavior on organizations’ ethical behavior using hierarchical regression analysis (Table 7) and structural equation modeling (Table 8).

**Table 7:** Hierarchical regression results

	Model 1	Model 2	Model 3
Block 1: controls			
Age	− 0.06**	− 0.02	− 0.02
Gender	0.00	− 0.01	0.00
Education—secondary school	− 0.01	0.00	0.00
Education—a bachelor’s degree	0.00	0.00	0.01
Education—master’s degree	0.02	0.02	0.03
Position—first level	0.41	0.04	0.04
Position—middle level	0.10***	0.11***	0.10***
Position—upper level	0.03	0.04	0.03
Organizational size—up to 100	0.10**	0.08*	0.09**
Organizational size—between 100 and 1000	0.05	0.04	0.05
Industry	− 0.03	− 0.03	− 0.02
Block 2: periods			
Economic circumstances—pre-crisis		0.05	0.03
Economic circumstances—after-crisis		− 0.07**	− 0.07**
Block 3			
Employee’s unethical behavior			− 0.26***
*N*	2024	2024	2024
R^2^	0.02	0.03	0.09
Model F	3.33***	4.34***	14.52***

Standardized regression coefficients are shown

*p < 0.05; **p < 0.01; ***p < 0.001

**Table 8:** A path analysis of employee’s unethical behavior on ethical behavior of organization

	*R* Square	Standardized coefficient (*β*)	Standardized coefficient (*β*)—Lower	Standardized coefficient (*β*)—Upper	C.R
Before crisis	0.11	− 0.33	− 0.23	− 0.43	− 6.65***
Crisis	0.08	− 0.29	− 0.18	− 0.40	− 6.54***
After crisis	0.18	− 0.43	− 0.32	− 0.53	− 10.23***

Dependent variable—ethical behavior of the organization

*p < 0.05; **p < 0.01; ***p < 0.001

Tables 7 and 8 indicate that employees’ unethical behavior significantly and negatively influenced the ethical behavior of organizations in three considered economic circumstances. The weakest effect was in crisis, while after the crisis, the negative influence increased. This supports Hypothesis 3.

As beta coefficients in Table 8 are close together, we calculated whether there are statistically significant differences between obtained beta coefficients in three different economic circumstances. The comparison of the standardized beta coefficients (Cumming, 2009) (1) before the crisis and in crisis revealed that the lower bound estimate for before crisis (− 0.23) exceeded the calculated cut-off value (− 0.34); hence, the difference between standardized beta coefficients for before the crisis and in crisis (Δ 0.04) was not statistically significant (*p* < 0.05). The standardized beta coefficients for (2) before the crisis and after crisis revealed that the lower bound estimate for after the crisis (− 0.32) exceeded the calculated cut-off value (− 0.37); therefore, the difference between standardized beta coefficients for before the crisis and after the crisis (Δ− 0.10) was not statistically significant (*p* < 0.05). Finally, the standardized beta coefficients for (3) crisis and after crisis indicated that the lower bound estimate for after crisis (− 0.32) exceeded the calculated cut-off value (− 0.34); thus, the difference between standardized beta coefficients for the crisis and after crisis (Δ− 0.14) was not statistically significant (*p* < 0.05). Calculations revealed that the effect of employee’s unethical behavior on organizational ethical behavior during three observed periods was not statistically different (*p* < 0.05). The difference between the standardized beta coefficients in crisis and after the crisis may be considered marginally significant, as the *p*-value was approaching significance (*p* < 0.1*: p* > 0.05) (Prischet et al., 2016).

## Discussion

The ethical behavior of organizations between 2006 and 2016 improved significantly, reflecting the findings supporting the continuous improvement of organizations’ ethical behavior (Kaptein, 2010) and engagement in CSR activities (Ryu, 2019; Williamson et al., 2006). Study results may have emerged because of the threat of unethical behavior to the reputation and financial results of the organizations (Kaptein, 2008; Paterson & Huang, 2019), society’s development, and increased expectations of various stakeholders regarding business ethics organization (Carroll & Buchholtz, 2008).

The significant and negative effect of employee’s unethical behavior on organizational ethical behavior was the weakest in the crisis, while after the crisis, it increased substantially, which is in line with previous theoretical presumptions about this relationship (Trevino et al., 2008, 2014). This study’s results may also indicate the “lag-effect” of crisis since explained variance and regression coefficients increased substantially after the crisis. This may be attributed to the heightened interest in organizational ethical behavior and increased sensitivity to unethical behavior in the crisis, which had skyrocketed the negative effect of employees’ unethical behavior on organizational ethical behavior after crisis time (Jacobs et al., 2014). The effect of employees’ unethical behavior on the ethical behavior of organizations supports the lag effect, as after the crisis, it was marginally significantly different from the effect in crisis (*p* < 0.1) (Pritschet et al., 2016). Accordingly, we may attribute the increased negative effect of employee’s unethical behavior on the business ethics of organizations (Trevino et al., 2008) after the crisis to the crisis and the “lag effect.” Additionally, a relatively large percentage of the variance explained in the ethical behavior of the organization (almost 20%), compared to the studies on the ethical behavior of organizations, which typically report small effect size of the considered predictors of ethical behavior, supported the importance of employees’ unethical behavior as a predictor of the ethical behavior of organizations. For instance, Moore et al. (2012) reported that the propensity to disengage morally explains up to 10% of the variance in unethical workplace behavior.

Employees’ unethical behavior had become significantly less acceptable in crisis. These findings may be rooted in numerous scandals and malpractices that occurred before the crisis during the transition period (Jacobs et al., 2014; Trevino et al., 2014). Moreover, higher acceptance of employees’ unethical behavior after the crisis, which did not return to the level before the crisis, demonstrates the substantial contribution of the economic crisis to the perception of employees’ unethical behavior. This may be attributed to increased individuals’ sensitivity to unethical behavior in the crisis and the visibility of unethical behavior through the media (Jelovac et al., 2011).

Among the control variables, the position of respondents in the organization and organizational size significantly affected organizational ethical behavior. Middle managers viewed the ethical duties of organizations as significantly less important compared to professionals in the survey. This corresponds to the findings that managers may have a “rosier view” of ethical issues compared to other employees (Trevino et al., 2008). Such a view of middle managers may also stem from middle managers’ primary focus on employees and organizations’ operational results. Next, the results showed that organizations with less than 100 employees engage significantly less in ethical behavior compared to organizations with more than 1000 employees, confirming the role of organizational size in business ethics of organizations (Enderle, 2004; Spence, 1999).

### Slovenian Context

We may argue that the crisis brought past unethical behavior into individuals’ minds, which was reflected in the lower acceptance of employees’ unethical behavior in the crisis due to the threat of unethical behavior. We based our assumptions on the following observations. In the 1990s, Slovenian managers had low ethical standards, which led to numerous scandals (Pučko, 2000). Well-integrated business ethics topics into the curricula of business schools in Slovenia can account for a steady improvement in employee’s unethical behavior in Slovenia also during the crisis (Duh et al., 2010).

Continuously striving to improve the ethical behavior of the organization, despite the crisis, reflects the organizational commitment to achieving sustainable and responsible goals and integrating ethical principles in organizations and their policies, strategies, and actions (Potocan et al., 2019). Moreover, tight connections with well-developed economies provide support for improving ethical behavior according to the EU’s legal responsibilities (Steurer & Konrad, 2009).

In general, decreased acceptance of employees’ unethical behaviors may reflect the findings of the survey from the late 1990s, which claimed that younger Slovenian managers consider unethical practices, like taking credit for another’s work, as less acceptable compared to their peers in Hong Kong and Australia (Pučko, 2000) and that Slovenian managers evaluate inappropriate business practices as more unethical compared to their peers in Russia, Turkey, and the USA (Hisrich et al., 2003).

Small organizations’ lower interest in ethical behavior may be attributed to their motives for the business establishment, typically job scarcity and low income (Duh et al., 2010; Mickiewicz et al., 2017). For such organizations, considering engagement in CSR beyond minimum standards is optional and costly (Williamson et al., 2006); thus, the concern about “ethical and CSR issues” is not the top priority (Steurer & Konrad, 2009).

### Implications

This paper has several theoretical implications. Our findings complemented previous knowledge about the importance of the predictors of individual, organizational, and situational behavior (Jones & Kavanagh, 1996; Umphress et al., 2010). This research outlines organizations’ ethical behavior and the employees’ unethical behavior over a longer period, considering their states in a crisis and comparing them to before and after the crisis. Prior studies have addressed behavior in organizations in favorable circumstances (Hisrich et al., 2003; Zuber & Kaptein, 2014), while fewer researchers have considered the behavior in a crisis (Jaffe & Tsimerman, 2011) or even over a longer period (Kaptein, 2010). Third, our empirical findings of the negative effect of employees’ unethical behavior on organizations’ ethical behavior complement the theoretical assumptions about this effect (Trevino et al., 2014) and provide the arguments for the lag effect of the organizations’ behavior in crisis.

The most significant practical implications of this research are the following. First, our results demonstrated the economic crisis’s influence on employees’ unethical behavior and organizations’ ethical behavior. This implies that managers, in light of the current economic crisis caused by COVID 19, should “actively manage” the ethical behavior of organizations. Since the tendency to improve organizations’ ethical behavior slowed down after the economic crisis, managers need to closely monitor their organizations and employees’ ethical behavior in the current crisis. Second, managers should raise the awareness of codes of ethics and conduct continuous in-service training to decrease the acceptability of unethical behavior in organizations (Kaptein, 2008). In the recruiting process, an additional set of criteria may be added to address the candidates’ view of unethical behavior to ensure their better fit with the organization’s ethical “standards” (Suar & Khuntia, 2010). Third, new generations of employees, those born after the 1980s (i.e., millennials), value earning money, possessing things, achieving success, and promoting the workplace at any cost (Remišová et al., 2019). Simultaneously, they have a low appreciation of ethically and socially responsible behaviors (Ng et al., 2010), implying that they are more prone to use unethical career advancement practices. In turn, managers need to establish suitable career advancement schemes to minimize unethical behavior due to the promotion.

## Limitations and Future Research

Our research has some limitations. First, the findings may have limited generalizability due to the specific Slovenian context, including the gradual transition approach and organizations’ self-management principles (Mrak et al., 2004). However, relatively similar societal and business contexts of former transition economies in Europe (Lang et al., 2013) broaden the implications of the results to former transition economies in Europe. The results may also be interesting for Western economies due to Slovenia’s traditionally strong linkages with the EU’s Western economies (Hisrich et al., 2003; Soulsby et al., 2017).

Second, peer-assessment of employees’ unethical behavior was used to reduce socially desirable response bias (Trevino et al., 2008; Zuber & Kaptein, 2014), but this can put into the forefront other biases as the peers might be considering their behaviors in their assessment of other employees’ behaviors (Ralston et al., 2009a). The ethical behavior of organizations was assessed using self-reports, reflecting the perceptions of the respondents working in the managerial and non-managerial positions, who may have a different opinion about ethics (Trevino et al., 2008). Nonetheless, this study focused on “organizational behavior according to goals of the ethical oriented activities of organizations,” which is a step beyond other studies that have relied on business professionals’ attitudes towards organizations’ duties (Furrer et al., 2010; Potocan et al., 2019).

Third, we used data from unrelated samples instead of cohorts typically used in longitudinal studies (Kish-Gephart et al., 2019). Although panel data could increase the data’s quality, access to the same respondents across different observations for a longer period is often not possible (Kaptein, 2010). Furthermore, even though each study had its limitations, conducting a series of studies may allow for stronger inferences to be made and more generalizable results to be obtained compared to a cross-sectional study on its own (Egri & Ralston, 2004). Fourth, this study addressed only a part of the spectrum of unethical behaviors, focusing on employees. Many other unethical behaviors of financers, customers, suppliers, and society were not addressed (Kaptein, 2008). Despite that, the 38-item instrument has been widely used in studies (Egri et al., 2000; Ralston et al., 2009a, 2009b).

In terms of future research, we can outline several directions. First, this survey should be replicated in different institutional, economic, political, cultural, and social settings to test whether the results are valid beyond the context considered in this survey. Second, it would be beneficial to examine further behavior in organizations in the COVID 19 crisis. This would help determine the magnitude of the effect of the current crisis on behavior in organizations compared to the effects of the economic crisis of 2009. Third, examining single items (Kaptein, 2010) measuring the latent variable of employees’ unethical behavior would help reveal the presence of individual unethical practices and the motives for each unethical practice (Remišová et al., 2019). Fourth, future research should compare employees in managerial and non-managerial positions in terms of unethical behavior and their perceptions of ethical behavior of organizations, since managers have a broader view of ethics compared to non-managerial employees (Trevino et al., 2008). Fifth, future research should pay attention to small business ethics (Spence, 1999). Finally, future replications could benefit from adding examples of unethical behavior related to financers, customers, suppliers, and society to broaden the spectrum of unethical behavior (Kaptein, 2008).
